# Recent findings and advancements in the detection of designer benzodiazepines: a brief review

**DOI:** 10.2478/aiht-2023-74-3771

**Published:** 2023-12-29

**Authors:** Dihua Wu, Li Fu

**Affiliations:** Hangzhou Dianzi University College of Materials and Environmental Engineering, Hangzhou, China

**Keywords:** adinazolam, clonazolam, deschloroetizolam, diclazepam, etizolam, flualprazolam, flubromazepam, flubromazolam, mass spectrometry, metabolism, phenazepam, pyrazolam, toxicology, adinazolam, deskloroetizolam, diklazepam, etizolam, fenazepam, flualprazolam, flubromazepam, flubromazolam, klonazolam, masena spektrometrija, metabolizam, pirazolam, toksikologija

## Abstract

This review article takes a closer look at a new class of psychoactive substances called designer benzodiazepines (DBZs) and the challenges of their detection. These are adinazolam, clonazolam, deschloroetizolam, diclazepam, etizolam, flualprazolam, flubromazepam, flubromazolam, phenazepam, and pyrazolam. They are central nervous system depressants and sedatives that can cause psychomotor impairment and increase the overdose risk when combined with other sedatives. DBZs undergo phase I and II metabolism similar to traditional benzodiazepines, but their specific metabolic pathways and the influence of genetic polymorphisms are yet to be clarified. Advances in liquid chromatography-tandem mass spectrometry (LC-MS/MS) have enhanced the method's sensitivity for DBZs and their metabolites in biological samples and coupled with improved blood sampling methods require less blood for drug monitoring. Further research should focus on elucidating their pharmacokinetic properties and metabolism in humans, especially in view of genetic polymorphisms and drug interactions that could inform clinical treatment choices. Even though we have witnessed important advances in DBZ detection and measurement, further refinements are needed to expand the scope of detectable DBZs and their metabolites. All this should help toxicological research to better identify and characterise the risks of chronic and polydrug abuse and facilitate clinical, forensic, and regulatory responses to this growing issue.

## Abstract (sl)

Svrha je ovoga preglednog članka dati bolji uvid u djelovanje nove skupine psihoaktivnih tvari zvanih dizajnerski benzodiazepini (DBZ) i u izazove njihova otkrivanja. Poimence, to su adinazolam, klonazolam, deskloroetizolam, diklazepam, etizolam, flualprazolam, flubromazepam, flubromazolam, fenazepam i pirazolam. Riječ je o depresivima i sedativima središnjega živčanog sustava koji mogu oslabiti psihomotornu funkciju te povećati rizik od predoziranja u kombinaciji s ostalim sedativima. DBZ-i prolaze fazu I. i II. biorazgradnje na način sličan onomu tradicionalnih benzodiazepina, no tek nam preostaje razjasniti njihove specifične metaboličke putove i kako na njih utječu genski polimorfizmi. Poboljšanja u tekućinskoj kromatografiji s tandemskom masenom spektrometrijom (engl. *liquid chromatography-tandem mass spectrometry*, krat. LC-MS/MS) povećala su osjetljivost metoda na DBZ-e i njihove metabolite u biološkim uzorcima, a poboljšanja u uzorkovanju krvi smanjila su količine potrebne za praćenje razina tvari. Ipak, potrebna su daljnja istraživanja koja će rasvijetliti njihova farmakokinetička svojstva i metabolizam u ljudi, napose u smislu utjecaja genskih polimorfizama i interakcija lijekova, što će pomoći pri odabiru kliničkog liječenja. Premda smo svjedoci važnih pomaka u otkrivanju i mjerenju DBZ-a, potrebna su daljnja poboljšanja kako bi se povećao opseg DBZ-a i njihovih metabolita koji se ovim metodama mogu otkriti. Sve to trebalo bi ne samo pridonijeti toksikološkim istraživanjima radi boljeg prepoznavanja rizika povezanih s kroničnom kombiniranom zloporabom droga nego i olakšati kliničke, forenzičke i regulatorne postupke kao odgovor na ovaj rastući problem.

Benzodiazepines are a class of psychoactive drugs that act on the central nervous system (CNS) by enhancing the activity of gamma-aminobutyric acid, the major inhibitory neurotransmitter in the brain (1). Since their introduction in the 1950s, benzodiazepines have been widely prescribed for treating anxiety, insomnia, seizures, and other disorders (2). Some common benzodiazepines include diazepam, alprazolam, clonazepam, and lorazepam.

Detection of benzodiazepines and their metabolites is important in therapeutic drug monitoring, forensic toxicology, and sports anti-doping testing (3). Traditionally, this had been done with immunochemical techniques like enzyme-linked immunosorbent assays (ELISA) (4), but immunoassays lack specificity and are open to interferences. Over the past two decades, they have been replaced by chromatography coupled with mass spectrometry (MS) (5), most notably gas chromatography-mass spectrometry (GC-MS) and liquid chromatography-tandem mass spectrometry (LC-MS/MS) (6, 7), as they are highly sensitive and specific enough to distinguish individual benzodiazepines and their metabolites.

The metabolism of benzodiazepines is complex and follows several pathways involving several enzymes. It starts with the phase I metabolism, which mainly involves oxidative reactions catalysed by cytochrome P450 (CYP) enzymes (8), most notably CYP3A4, CYP2C19, and CYP2B6 (9, 10). Follow the phase II conjugative reactions like glucuronidation mediated by uridine 5′-diphospho-glucuronosyltransferase (UGT) enzymes (11). High variability in benzodiazepine metabolites poses certain challenges to their detection and therapeutic monitoring, but recent advances in the understanding of benzodiazepine metabolism pathways have paved the way for developing more sensitive and selective detection methods, such as radiolabelling and high-resolution mass spectrometry (12), which have discovered benzodiazepine metabolism by intestinal microbiota and previously undetected circulating and excreted metabolites (13). Further discoveries of differences in benzodiazepine biotransformation between humans and preclinical animal models (14) have shed new light on the extrapolation of pharmacokinetic data from animal studies to humans, whereas new *in vitro* tools like recombinant CYPs (15), human liver microsomes (16), and hepatocytes (17) have facilitated detailed metabolism studies of benzodiazepines. Alongside these advances in metabolism research, analytical methods for benzodiazepine detection continue to be refined and upgraded. The ongoing developments in this area will advance therapeutic drug monitoring, toxicological analysis, and medication safety of this important class of psychoactive drugs.

Designer benzodiazepines (DBZs) are compounds derived from traditional benzodiazepines, featuring slight chemical modifications to achieve specific pharmacological effects or circumvent existing drug regulations. Unlike their well-studied counterparts, these designer variants have often escaped comprehensive clinical testing, so their safety profiles and potential risks are unclear. While traditional benzodiazepines are controlled and prescribed, DBZs may fall outside strict legal regulation, raising concerns about their misuse and safety. The aim of this review is to take a look at the toxicology of DBZs and the challenges of their detection, considering the recent advances and new detection techniques that can improve clinical practice, forensic toxicology, and drug safety monitoring of this widely used class of psychoactive drugs.

## TOXICOLOGY AND PUBLIC HEALTH RISKS

**Adinazolam** is a triazolobenzodiazepine of lower cardiac toxicity than other antidepressants like amitriptyline, as it does not significantly affect intraventricular conduction or cause arrhythmia, as observed in canine studies of post-myocardial infarction (18). Neuropharmacologically, adinazolam reduces norepinephrine and serotonin release in the hippocampal CA1 region, which may contribute to its anxiolytic and antidepressant properties (19). In elderly populations, the drug and its metabolite show reduced clearance, which, in turn, may lead to poorer psychomotor performance and cognitive impairment (20). Its profile is similar to atypical antidepressive and anxiolytic drugs that induce “sleep slow wave” EEG patterns at moderate and high doses (21). Adinazolam's antidepressant activity may be mediated by heightened serotonin neurotransmission in rat hippocampal pyramidal neurons (22). Its sedative and psychomotor effects are owed to its metabolite, N-demethyladinazolam (NDMAD), rather than the parent drug (23).

Being a designer drug, adinazolam is associated with growing public health concerns, especially in fatal and drug-related cases (24). At a dose of 30 mg, adinazolam can impair psychomotor and cognitive performance, and this effect is exacerbated when combined with ethanol, although a synergistic effect is not strongly supported (25). One study (26) reported that both adinazolam and its metabolite effectively induced sedation, psychomotor impairment, and memory impairment in healthy participants who were receiving 10 mg doses of adinazolam.

**Clonazolam**, the triazolo-analogue of clonazepam, was first synthesised in the 1970s and emerged as the most potent benzodiazepine with hypnotic, anxiolytic, sedative, muscle relaxant, and anticonvulsant properties (27). Prescribed quantities span from 0.5 mg (for non-addicted individuals) to 4 mg (for habitual benzodiazepine users). Sommerfeld-Klatta et al. (28) documented a case of a 26-year-old woman deliberately ingesting 10 mg of clonazolam powder. Despite the substantial dose and toxic blood levels, the patient's response was confined to an extended state of unconsciousness, reduced muscular tonicity, and impaired reflexes. Respiratory failure and cardiovascular complications, typically associated with benzodiazepine overdoses, were notably absent. The authors conclude that despite its high potency clonazolam is relatively safer in cases of acute overdose than other benzodiazepines.

**Deschloroetizolam**, classified as a short-acting thienotriazolodiazepine due to the absence of a chlorine substitution on its benzene ring, is a less potent analogue of etizolam. It was first reported by the UK Focal Point after the analysis of a seized blue tablet in September 2014 (29).

In a case reported by Balkhi et al. (30), the corpse of a 31-year-old male drug abuser was found with injection materials and small plastic bags containing various DBZs, including deschloroetizolam. Autopsy revealed multi-organ congestion, likely of toxic origin, with no evident natural diseases or traumatic lesions related to death. The time between death and discovery was estimated to be less than 48 hours. Analysis of femoral blood and urine samples, collected during autopsy, revealed deschloroetizolam at a concentration of 11 μg/L in the femoral blood and a concentration below the limit of quantification in urine.

Another study involved a researcher who self-experimented with 6 mg of deschloroetizolam (31). To monitor the drug's presence, he used the NeoSal device to gather saliva samples over 18 hours. The drug reached the highest concentration at 30 minutes, followed by a rapid decrease, but traces remained for the entire 18 hours. This decrease correlated with the quick onset and subsequent decline in the volunteer's physical symptoms, which included tiredness, dizziness, difficulty speaking, and problems with concentration.

**Diclazepam**'s initial documentation dates back to a German report in August 2013 (32). Three patients were received by the emergency department over poisoning with diclazepam, in two cases combined with stimulants and dissociatives. Patients manifested agitation, disorientation, CNS depression, and withdrawal symptoms. A recent investigation (33) used a combination of nuclear magnetic resonance (NMR), LC-MS/MS, GC-MS, and self-experimentation to delve into the pharmacokinetics and metabolism of diclazepam within the human body. Diclazepam exhibited an approximate elimination half-life of 42 hours and underwent metabolic conversion into active benzodiazepines such as delorazepam, lorazepam, and lormetazepam. These metabolites could be detected in urine for 6–19 days post-ingestion, while unmetabolised diclazepam could be identified in blood serum for up to 99 hours. The study also showed that standard immunochemical drug tests were not sensitive enough to identify low doses of diclazepam. In fact, all serum samples tested negative for diclazepam metabolites (delorazepam, lorazepam, and lormetazepam), despite the assay having a sufficient cross-reactivity of 72 %. LC-MS/MS, in turn, could detect its metabolites for nearly three weeks post-ingestion. This comprehensive study underscores diclazepam's long activity and challenges in detection with conventional techniques.

**Etizolam**, a thienodiazepine akin to benzodiazepines in its effects, has been a prescription drug in a number of countries for over 35 years. In therapeutic dosages ranging from 0.5 to 3 mg per day, etizolam elicits pharmacological responses and exhibits a safety profile parallel to those of conventional benzodiazepines such as diazepam (34). Reports of harmful effects are mostly related to its non-medical application, particularly the ingestion of illegal pills containing varying doses of etizolam and other unidentified substances (35). In such settings, etizolam is often combined with opioids, alcohol, and other CNS depressants, which significantly heightens susceptibility to strong adverse reactions, including respiratory depression. Instances of lethal overdoses almost invariably involve a complex interplay of polydrug toxicity, with etizolam seldom standing as the sole culprit (36).

**Flualprazolam** is an analogue of alprazolam with a fluorene moiety, which was first reported to the European Monitoring Centre for Drugs and Drug Addiction (EMCDDA) by Swedish police in January 2018 (37). A more recent report from California about traffic accidents related to driving under the influence of flualprazolam between 2018 and 2020 (38) shows that motorists with relatively low flualprazolam blood concentrations, averaging 12 ng/mL and ranging between 5.5 and 26 ng/mL, exhibited CNS depression consistent with benzodiazepine usage and were weaving between lanes. This account warns that flualprazolam can considerably impair driving ability even at concentrations below 10 ng/mL, which is much lower than anticipated for other benzodiazepines. Kriikku et al. (39) reported median blood flualprazolam concentration of 18.0 ng/g in 33 post-mortem cases and observed that Swedish victims were significantly older than the Finnish. Polydrug use, especially of flualprazolam and opioids was common, and flualprazolam was determined as the cause of death in 13 cases.

**Flubromazepam** originates from the 1960s, but became widespread as recreational drug only in 2012. It was retailed by online vendors to be used in formulations of psychoactive blends. Its primary metabolic pathway is hydroxylation, most likely at position 3 of the molecule, leading to 3-hydroxy-flubromazepam, apparently catalysed by CYP450 enzymes (40). In terms of pharmacokinetics, flubromazepam has a prolonged action and elimination half-life of 106 hours, similar to other long-acting benzodiazepines like diazepam and nordazepam. Moosmann et al. (40) report that a 4 mg dose reached its highest serum concentration of approximately 78 ng/mL six hours after intake and that its monohydroxylated metabolites remained detectable in serum for up to 23 days and in urine for up to 28 days. Cases admitted to emergency departments were described as experiencing severe agitation, delirium, and CNS depression. Fatalities linked directly to flubromazepam are scarce, and more common are those attributed to other co-administered substances (41).

**Flubromazolam** stands out for its pronounced potency. A mere 0.5 mg dose, resulting in serum concentrations peaking between 8 and 10 ng/mL (42) can cause sedation. This compound can be found in urine for up to 6.5 days, while its hydroxylated metabolite remains traceable for up to 8 days following a single administration (43). Its potency, long life, and easy accessibility make flubromazolam particularly alluring to abusers. Abdul et al. (44) report that flubromazolam in post-mortem cases ranged from 1 to 70 mg/L, which is noteworthy, as no range has been established that elicits specific clinical manifestations. All samples, the authors also noted, showed several other sedatives, including heroin and other benzodiazepines. They emphasised the importance of including flubromazolam in post-mortem screens due to its potency and potential contribution to fatalities, especially given the growing prevalence of new psychoactive substances in toxicology.

**Phenazepam** was designed in the Soviet Union in the 1970s as an anxiolytic and anticonvulsant but has become a popular recreational drug in Western countries in recent times. In comparison to benzodiazepines, phenazepam is more potent, with effects persisting for up to three weeks (45). Recreational doses range from 2 to 10 mg, and blood levels between 200 and 400 μg/L have been associated with functional impairments and death (46, 47). For instance, the first UK death directly involving phenazepam was reported in July 2011, followed by a second in November 2011 (48). These incidents underscored the potential for severe adverse health consequences of the recreational use of the drug.

**Pyrazolam** is a benzodiazepine derivative originally developed in the 1970s (49) and first reported to the EMCDDA in August 2012 (50). Moosmann et al. (51) profiled pyrazolam using NMR spectroscopy and LC-MS/MS. One of the authors ingested a 1 mg dose of and gave serum and urine samples across several days to establish its pharmacokinetic behaviour. The analysis showed an elimination half-life of about 17 h, as opposed to 6 h claimed by the online vendor. The parent compound remained detectable in the serum for over 50 h, and in urine for up to 6 days. The absence of metabolites, furthermore, suggests that pyrazolam does not biotransform in the body as much as other analogous benzodiazepines. Lehmann et al. (52) detected it in various body fluids and tissues, which indicates widespread distribution in the body after ingestion. Its concentration varied from 11 μg/L in pericardial fluid to as high as 500 μg/L in urine.

## RECENT ADVANCES IN ANALYTICAL METHODS TO DETECT DBZS AND THEIR METABOLITES

### Advances in LC-MS/MS

LC-MS/MS has become the gold standard for benzodiazepine and metabolite analysis in biological samples. Recent advances have improved its sensitivity, selectivity, and throughput. Bergstrand et al. (53) developed a method for urine analysis of 11 DBZs that has only a 3.1 min run time. The method is sensitive (with the detection limit of 1–10 ng/mL), accurate, precise, and selective. Mastrovito et al. (54), in turn, developed an LC-MS/MS method preceded by liquid-liquid extraction, able to detect and measure 12 DBZs and their metabolites in blood. It has good validation results and has detected DBZs in 70 % of samples that were confirmed negative in the second-step screening.

In addition, advanced methods improve DBZ separation from sample matrices and have faster runs. Behnoush et al. (55) compared conventional high and ultra-high performance liquid chromatography (UHPLC). UHPLC had 25 min shorter run time, lower solvent consumption, 5–10 times better detection limits, better resolution, sensitivity, and efficiency. Vårdal et al. (56) developed a method using parallel artificial liquid membrane extraction coupled with UHPLCMS/MS. Using less organic solvent, this method had higher throughput, better sensitivity, and reproducibility on small blood volumes.

On-line SPE-LC-MS/MS workflows integrate solid-phase extraction directly with LC, providing automated sample cleanup. Turbulent flow chromatography is also applied for rapid online extraction prior to LC-MS (57). Such approaches minimise sample handling while maximising detection sensitivity and accuracy. Improving analytical sensitivity can help detect metabolites at low concentrations, which can be achieved by optimising chromatographic conditions, selecting appropriate internal standards, and using selected (multiple) reaction monitoring. To that end, Racamonde et al. (58) have developed an online SPE-LC-MS/MS method for determining 23 DBZs and their metabolites in 100–200 mL wastewater samples using automated extraction with Oasis MCX cartridges and LC-MS/MS analysis with multiple reaction monitoring. The reported limits of quantification range from 0.1 to 18 ng/L and the method shows good recoveries and precision.

Nissilä et al. (59) report improved efficiency of electrospray ionisation – mass spectrometry (ESI-MS) if a new nanoelectrospray ionisation is used. Mass spectrometer sensitivity can be boosted with optimised ion optics, ion transfer, and vacuum systems. Coupling to the high-field asymmetric waveform ion mobility spectrometry (FAIMS) improves the selectivity of MS by separating ions based on mobility differences (60). Mass spectrometry can also run faster for high-volume benzodiazepine testing if the RapidFire technology is used, which enables quick online SPE (61, 62). In addition, multiple analytes can be determined in a single injection with multiplexing based on different isotope labelling or mass defect separation (63, 64). Multiplexing improves throughput while minimising analytical variability.

### New immunochemical techniques

While LC-MS/MS remains the gold standard (65), immunoassays can find niche applications owing to their simplicity of use and cost-effectiveness. Recent advances with novel antigens and formats seem to improve their specificity. For example, hybridoma technology can produce monoclonal antibodies targeting specific benzodiazepine metabolites like oxazepam glucuronide and temazepam, which renders them superior to polyclonal antibodies used in traditional immunoassays (66).

As for sensitivity, Darragh et al. (67) reported that the hydrolysis-enhanced CEDIA^TM^ immunoassay was superior to traditional immunoassays but still missed 22 % of samples found positive by LC-MS/MS. It did not detect lorazepam and other benzodiazepines primarily excreted as glucuronides.

While some innovative immunoassays, such as enzyme-multiplied immunoassay technique (EMIT) relying on luciferase, greatly improve sensitivity (reaching the pg/mL range), Bertol et al. (68) report issues with EMIT benzodiazepine detection in urine owed to high cross-reactivity for some compounds, which leads to greatly overestimated concentrations and risk of false positives.

### Alternative sampling methods

One avenue of improving DBZ detection and measurement is by simplifying blood collection. Dried blood spot (DBS) microsampling requires smaller volumes of capillary blood (20–50 μL) collected onto filter paper than standard blood draws and can be used for sensitive LC-MS/MS, as reported by Moretti et al. (69), who found good sensitivity, accuracy, and precision in a LCMS/MS using DBS for 27 benzodiazepines and their metabolites. Most analytes remained stable in DBS for three months.

Volumetric absorptive microsampling (VAMS), in turn, is a technique for collecting small, precise volumes of blood or other biological fluids using an absorbent tip allowing easier and more convenient sampling, storage, and transport for analysis. Mestad et al. (70) validated several common classes of drugs of abuse, including benzodiazepines using VAMS. All benzodiazepines achieved extraction recoveries above 70 %.

### Metabolite detection and identification

New high-resolution accurate-mass spectrometers like quadrupole time-of-flight (QToF) and Orbitrap make it possible to identify metabolites and their isomers in very low concentrations. Further support to identifying biotransformation pathways is provided by *in silico* prediction tools. For instance, Gundersen et al. (71) screened blood samples taken post-mortem using a UHPLCQTOF-MS against a database of 374 new psychoactive substances. They identified five in two samples and 35 potentially new substances, but only phenibut was considered a plausible finding.

Because DBZs can be surreptitiously added to alcoholic beverages like beer to incapacitate a victim, making them more vulnerable to assault or theft, Yao et al. (72) developed a polymer monolithic microextraction method using poly(*N*-vinylcarbazoleco-divinylbenzene) and analysed six DBZs in spiked beer and urine samples with UHPLC/Q-Orbitrap MS. Recoveries ranged from 79.6 to 95.2 %. The detection limits were below the thresholds set by the United Nations Office on Drugs and Crime (UNODC).

## CONCLUSIONS AND FUTURE DIRECTIONS

Recent findings provide initial insights into the clinical effects of DBZs and related toxicological risks. All DBZs exhibit sedative-hypnotic properties, impair the psychomotor function, and cause CNS depression, but their potency and duration of effects vary substantially. What is clear, though, is that their co-administration with other CNS depressants significantly elevates the risk of serious adverse events.

However, their pharmacokinetic properties and metabolism in humans have yet to be elucidated. Preliminary data indicate metabolic pathways analogous to traditional benzodiazepines, but we still need to identify specific enzymes involved in their biotransformation. In this respect, knowing more about the contribution of genetic polymorphisms and drug interactions could inform clinical treatment choices.

Another avenue of progress concerns advances in DBZ detection and measurement. There we see much more progress, but further refinements are needed to expand the scope of detectable DBZs and their metabolites.

Further toxicological research should also characterise the risks of chronic and polydrug abuse to facilitate clinical, forensic, and regulatory responses to this growing issue.
